# Our senile case with total crescentic massive atheromatous calcification in thoracic aorta

**DOI:** 10.1186/1749-8090-8-S1-P50

**Published:** 2013-09-11

**Authors:** U Yetkin, I Yurekli, A Gurbuz

**Affiliations:** 1 Izmir Katip Celebi University Ataturk Training and Research Hospital, Department of Cardiovascular Surgery, Izmir, Turkey

## Background

Thoracic aortic segments are the most commonly involved localizations of intimal vascular calcifications that are usually assumed to be due to atherosclerosis. Etiological factors include chronic renal insufficiency, diabetes, hypertension and atherosclerosis that all cause endothelial damage.

## Method

Our case was an 89-year-old female. She was under our outpatient follow-up due to hypertension, senile diabetes and hypertriglyceridemia. Her electrocardiogram revealed no pathological finding. She had diabetes for 10 years which was regulated with oral antidiabetics with a fasting serum glucose level of 141 mg/dl. Her total serum cholesterol and triglyceride levels were 192 and 333 mg/dl, respectively. Her renal function tests were consistent with her age. Her hematocrit level was 31.7%.

## Results

Her chest x-ray showed total crescentic massive atheromatous calcification in aortic knob. Transthoracic echocardiography measured left ventricular end-systolic/end-diastolic diameters as 30/45 mm with an ejection fraction of 55%. Diffuse atheromatous calcification of the aortic wall and mild aortic and mitral regurgitation were the remaining findings. She was discussed about in our surgical council and medical ambulatory follow-up with regulation of the co-morbid factors by related clinical branches was the decision. Dietary regulations and antiaggregant therapy were emphasized and outpatient follow-up with 3-month intervals was recommended.

## Conclusion

Atheromatous calcification of the thoracic aortic wall was used to be evaluated as a passive degenerative process of intimal and medial layers. But, recently it is believed to be an active programmed process. Chronic inflammation as the chief role player of the atherosclerotic process and the personal immunity are considered as the main factors in calcification. As in our case, multidisciplinary control of co-morbid factors and frequent outpatient follow-up are necessary.

